# Analysis of Polymorphisms Associated with Base Excision Repair in Patients Susceptible and Resistant to Noise-Induced Hearing Loss

**DOI:** 10.1155/2019/9327106

**Published:** 2019-11-14

**Authors:** Enmin Ding, Jiadi Guo, Xin Ge, Rongjian Sheng, Jian Chen, Hengdong Zhang, Baoli Zhu

**Affiliations:** ^1^Jiangsu Provincial Center for Disease Prevention and Control, Institute of Occupational Disease Prevention, Nanjing, Jiangsu Province, China; ^2^Center for Global Health, School of Public Health, Nanjing Medical University, Nanjing, Jiangsu Province, China; ^3^Yizheng Hospital, Drum Tower Hospital Group of Nanjing, Yizheng, Jiangsu Province, China

## Abstract

**Objective:**

Noise-induced hearing loss (NIHL) is one of the most common occupational health risks in both developed and industrialized countries. It occurs as a result of interactions between genetic and environmental factors. Nevertheless, inherited genetic factors contributing to NIHL are not well understood. Therefore, we aim to investigate whether genetic mutations in three important base excision repair genes (*OGG1*, *APEX1*, and *XRCC1*) may influence susceptibility to NIHL.

**Methods:**

Three SNPs in *OGG1*, *APEX1*, and *XRCC1* were genotyped from 1170 noise-exposed workers and were classified into 117 most susceptible and 117 most resistant individuals.

**Results:**

Results showed that the rs1799782 TT genotype located in the *XRCC1* coding region and rs1130409 GG/GT in the *APEX1* coding region were associated with increased risk for NIHL in a Chinese population. Compared to the rs1799782 C allele frequency, the T allele frequency was increased in the sensitive group (adjusted OR = 1.51, 95%CI = 1.01 to 2.26, *P* = 0.043). The rs1130409 G allele frequency was also increased in the sensitive group compared to the resistant group (adjusted OR = 1.59, 95%CI = 1.10 to 2.31, *P* = 0.015). Moreover, rs1130409 and drinking had a statistically significant interaction (*P* = 0.0002), while rs1799782, rs1130409, and smoking also had a statistically significant interaction (*P* < 0.0001).

**Conclusions:**

*XRCC1* rs1799782 and *APEX1* rs1130409 may have potential as biomarkers for the screening of susceptibility to NIHL in workers exposed severe noise.

## 1. Introduction

Noise-induced hearing loss (NIHL) has been the second most common form of severe sensorineural hearing impairment, besides age-related hearing loss (ARHL). It is one of the leading occupational diseases both in developed and industrialized countries [1].

NIHL is a complex disease, caused by interactions between genetic and environmental factors, with large differences in hearing loss occurrence after similar noise exposure [2, 3]. This interindividual variability has been considered to be due to interactions between genetic and environmental factors, as well as living habits. It is believed that besides noise, ototoxic substances, heat, vibrations, and individual factors such as age, smoking, and blood pressure have an effect on the development of NIHL [4]. Numerous variations in susceptibility to NIHL have been reported. Single nucleotide polymorphisms (SNPs) are known as the most common form of genetic variation in the mammalian genome, with about 15 million SNPs found among all humans. So far, SNPs in genes such as *FOXO3*, *DNMT*, *HSP70*, *CAT*, *Notch*, and *KCNQ4* have been identified in many association studies regarding NIHL involving human subjects [5–10].

DNA repair is the most important defense mechanism against DNA lesions, which are caused by environmental factors and normal metabolic activity in humans [11]. DNA damage is identified and processed by a variety of distinct pathways collectively called the “DNA damage response (DDR)” pathways [12]. DDR includes mechanisms such as direct repair (DR), mismatch repair (MMR), double-strand break repair (DSBR), nucleotide and base excision repair (NER and BER), and DNA interstrand crosslink repair [13, 14]. BER, a key mechanism of the DNA repair pathway, mainly plays a role in repairing damage to single bases in DNA molecules. BER is the main guard against DNA damage as a result of both normal and abnormal cellular metabolism, including methylation, deamination, hydroxylation, reactive oxygen radicals, and physical and chemical factors (such as X-rays and alkylating agents) [15]. Moreover, the BER pathway is the primary mechanism that defends against oxidative stress-induced DNA damage in cells. BER is known to act on small DNA lesions or modified bases to repair damage by removing and replacing damaged base pairs. Enzymes involved in BER include human 8-oxoG DNA glycosylase1 (*hOGG1*), apurinic/apyrimidinic endonuclease 1 (*APE1* or *APEX1*), and the X-ray repair cross-complementing group 1 (*XRCC1*). Variations that occur in BER-related gene regions can lead to abnormality of repair functions, increasing the probability of developing diseases [16].

Numerous studies have reported on the association of genetic factors, including DNA synthesis-related genes, DNA repair pathways, cell cycle control, and apoptosis, with NIHL individual susceptibility of workers exposed to industrial noise. Shen et al. showed that the *APEX1* rs1130409 and *hOGG1* rs1052133 polymorphisms contribute to the susceptibility of NIHL in Chinese populations [17, 18]. However, this conclusion is slightly controversial, as the study included an insufficient number of samples compared to the study conducted by Konings et al. [8], which included 1261 Swedish and 4500 Polish, noise-exposed labourers. Moreover, the association between polymorphisms of the *XRCC1* gene and NIHL susceptibility was not reported before.

In this study, we aim to investigate whether BER genes are associated with susceptibility to NIHL in 117 sensitive and 117 resistant individuals selected from a cohort of 1170 noise-exposed workers. By using the Single Nucleotide Polymorphism Database (dbSNP data), three putative SNPs in *hOGG1* (rs2072668), *APEX1* (rs1130409), and *XRCC1* (rs1799782) were selected and the genetic interactions of these three polymorphisms and their relation to NIHL risk among the Northern Han Chinese population were evaluated.

## 2. Patients and Methods

### 2.1. Patients

A total of 1170 noise-exposed workers from a single factory located in northern China were enrolled in the current study in December 2017. Informed consent was obtained from all individual participants, and research was approved by the ethical committee of the Jiangsu Provincial Center for Disease Prevention and Control. Patient data, including general information, lifestyle, past medical history, and exposure to chemical/physical factors, was gathered. To exclude confounding factors other than genetic susceptibility as much as possible, out of the 1170 workers, we selected the 10% which were most susceptible and most resistant to noise, respectively. The 10% most resistant and the 10% most sensitive subjects were selected using the HTL at 3 kHz as a measure of noise susceptibility [8].

### 2.2. Pure Tone Audiometry and Environmental Noise Measurement

As described in a previous study [17], 500, 1000, 2000, 3000, 4000, and 6000 Hz pure tone air hearing threshold tests were conducted in a sound-attenuating chamber by an otolaryngologist. The subjects were required to avoid loud noise exposure (>85dB) for at least 12 hours prior to the pure tone audiometry. An ascending method in 5 dB(A) steps was adopted to ascertain the hearing threshold levels of both ears according to the Diagnostic Criteria of Occupational Noise-Induced Hearing Loss of China [10].

Individual sound pressure noise meters (Noise-Pro, Quest, Oconomowoc, WI USA) were used to measure noise exposure levels for each individual in the workplace at 10 a.m., 3 p.m., and 5 p.m. for three consecutive days.

### 2.3. SNP Selection

For the aim of the current study, the analysis of the genotyping data was focused on candidate SNPs located in genes involved in the base excision repair pathway. First, SNPs were selected based on the data of the 1000 Genomes Project and dbSNP (https://www.ncbi.nlm.nih.gov/), as well as a primary literature review. The criteria for identifying SNPs included a minor allele frequency (MAF) in the Han Chinese population (CHB) of >0.10 and the linkage disequilibrium (LD) *r*^2^ > 0.8. Following that, we screened out the SNPs which were located in functional regions of the genes (missense, 3′UTR, and 5′UTR) or were previously reported to be involved in human diseases. Finally, rs2072668, rs1130409, and rs1799782 met our requirements and were used for subsequent experiments.

### 2.4. SNP Genotyping

Genomic DNA was extracted from 200 *μ*L of peripheral blood samples using the QIAcube HT and QIAamp 96 DNA QIAcube HT Kits (Qiagen, Dusseldorf, Germany). The three SNPs, rs2072668, rs1130409, and rs1799782, were genotyped using the ABI TaqMan SNP genotyping assay on the ABI 7900HT system (Applied Biosystems, Foster City, CA, USA). The genotyping results were analyzed using the ABI SDS 2.4 Software (Applied Biosystems).

### 2.5. Statistical Analysis

The chi-square goodness-of-fit test was used to evaluate the deviation of the genotype frequencies of the three SNPs from the Hardy-Weinberg equilibrium (HWE) in the 10% most sensitive subjects. Comparison of the distribution of the *hOGG1*, *APEX1*, and *XRCC1* genotypes between sensitive and resistant individuals was conducted using Pearson's chi-square test. Multivariate unconditional logistic regression adjusted for age, sex, tobacco use, and alcohol consumption was performed to estimate the odds ratio (OR) and 95% confidence interval (95% CI) for the associations of the selected SNPs with NIHL risk. Generalized multifactor dimensionality reduction (GMDR), a generalized combinatorial approach for detecting gene-by-gene and gene-by-environment interactions, adopts dimension reduction strategy to discover interactions [19]. GMDR v0.9 software was used to explore the interactions of the three selected SNPs with environmental factors. All statistical analyses were performed using the SPSS 24.0 software (IBM, NYC, USA), and values of *P* < 0.05 were considered statistically significant.

## 3. Results

### 3.1. Demographic Characteristics of the Study Subjects and the Hardy-Weinberg Test

General demographic and lifestyle features (age, sex, tobacco, and alcohol consumption habits), duration of noise-exposed work time, noise intensity, and high-frequency hearing threshold of the sensitive and resistant groups are shown in Table 1. There was no significant difference between sensitive and resistant subjects regarding general characteristics and lifestyle features, duration of noise-exposed work time, and noise intensity (*P* > 0.05). However, the average high-frequency hearing threshold was significantly higher in the sensitive group (52.35 ± 6.63 dB) than the resistant group (8.98 ± 2.27 dB) (*P* < 0.001). General data of the selected SNPs and the Hardy-Weinberg test results are shown in Table 2. Rs2072668 of *hOGG1*, rs1799782 of *XRCC1*, and rs1130409 of *APEX1* are intron, missense, and missense variants, respectively. All selected SNPs have minor allele frequencies ≥ 5% and are within the Hardy-Weinberg equilibrium (HWE) (*P* > 0.05).

### 3.2. Single SNP Analysis

Table 3 shows genotype frequencies of the sensitive and resistant groups. The *P* values resulting after statistical analysis of the single SNPs were also presented. In the codominant model, rs1799782 TT, rs1130409 GG, and rs1130409 GT were shown to be more frequent in the sensitive group (*P* = 0.005, OR = 8.92, 95%CI = 1.91 to 41.63; *P* = 0.039, OR = 2.21, 95%CI = 1.04 to 4.70; *P* = 0.004, OR = 2.48, 95%CI = 1.34 to 4.61, respectively). For the rs1130409 dominant model, genotypes GG and GT were found to be more frequent in the sensitive group (76.1%) compared to the resistant group (58.1%) (*P* = 0.003, OR = 2.39, 95%CI = 1.34 to 4.27). Genotype TT was more frequent in the sensitive group (12.0%) compared to the resistant group (1.7%) in the rs1799782 recessive model with an OR of 8.83 (*P* = 0.005, 95%CI = 1.93 to 40.36). Furthermore, compared to the rs1799782 C allele frequency, the T allele frequency was shown to be increased in the sensitive group (*P* = 0.046, OR = 1.51, 95%CI = 1.01 to 2.26). In addition, the rs1130409 G allele frequency was also increased in the sensitive group compared to the resistant group (*P* = 0.015, OR = 1.59, 95%CI = 1.10 to 2.31).

### 3.3. Stratification Analysis

Stratified analyses of SNPs were conducted under the allelic model, and the results were presented in Table 4. An increased risk was evident in individuals with more than 95 dB(A) cumulative noise exposure who carried the *XRCC1* rs1799782 T allele (adjusted OR = 1.76, 95%CI = 1.05 to 2.98).

### 3.4. Gene and Environment Interaction Analysis

**Figure 1:**
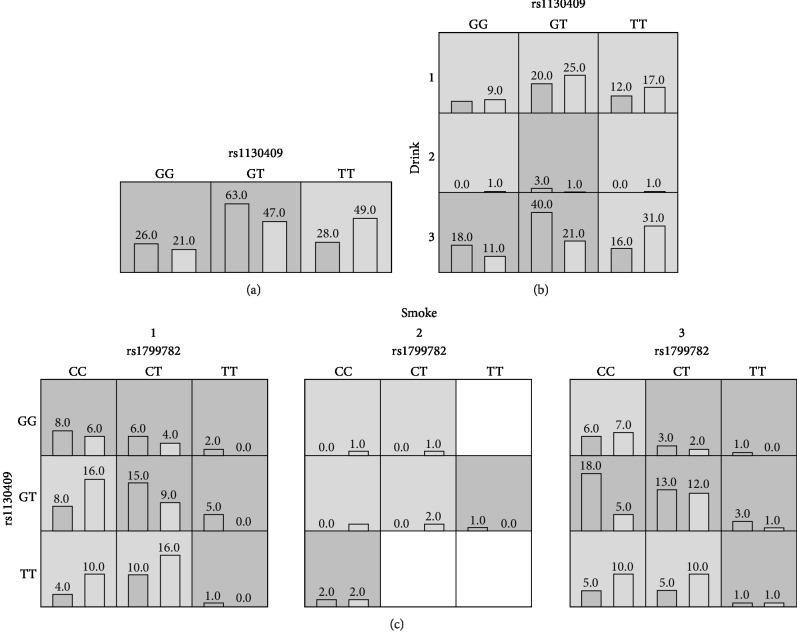
The best fit model gained by the analysis of GMDR. The implications of bars and background color in each multifactor cell are as follows. The left bars represent the sum of scores in the case and the right represents the control. High-risk cells are expressed by black shadow if the ratio of the number of cases to the number of controls exceeds the preset value *T*, as low-risk cells by light shadow if not more than the threshold and empty cells by no shadow which means no cases and controls. The multifactor cells labeled as “high risk” or “low risk” are then used to assess the classification and prediction accuracy, thus identifying the best model in the subsequent steps (drink 1: now, 2: ever, 3: never; smoke 1: now, 2: ever, 3: never).

We used the GMDR v0.9 software to detect the interaction of the three selected SNPs with environmental factors.  Table 5 shows the best fit model, testing balanced accuracy, cross-validation (CV) consistency, and *P* values obtained. In all models, rs1130409, rs1130409-drinking, and rs1799782-rs1130409-smoking were the best fit models. The analysis showed that rs1130409 and drinking had a statistically significant interaction (*P* = 0.0002, OR = 2.77, 95%CI = 1.61 to 4.77). Rs1799782, rs1130409, and smoking also had a statistically significant interaction with *P* < 0.0001 (OR = 3.71, 95%CI = 2.16 to 6.38). Diagrams of the best fit model are shown in Figure 1.

## 4. Discussion

Our results showed a statistically significant association of the rs1799782 TT genotype located in the *XRCC1* coding region and the rs1130409 GG/GT in the *APEX1* encoding region with an increased risk of NIHL in a Chinese population. Notably, the *APEX1* rs1130409 polymorphism has been previously reported to contribute to the susceptibility of NIHL in an Eastern Chinese population by Shen et al. Thereby, our results provide additional evidence that *APEX1* rs1130409 is a potential gene involved in NIHL susceptibility. Moreover, rs1799782 *XRCC1* was shown to be associated with NIHL susceptibility in a Chinese population for the first time.

*XRCC1* is a 33 kb long gene located in the chromosome 19q13.3 region. It consists of 17 exons and encodes a 2.2 kb transcript, producing the X-ray cross-complementing group 1 protein. It has potential interactions with DNA polymerase-*β* (POLB), poly ADP ribose polymerase (PARP), and DNA ligase III in the BER pathway. The rs1799782 (Arg194Trp, 580C>T) mutation within the *XRCC1* gene leads to a change in amino acids. These changes may alter the efficiency of XRCC1 in DNA repair and may have vital functional significance. Previously published research showed that the *XRCC1* gene codon 194 (rs1799782) is located at a conserved residue in the human genome, indicating that this polymorphism may have functional significance [20]. Moreover, there are studies that suggest that protein function can be affected by amino acid substitutions in evolutionarily conserved regions [21]. However, the functional effect of *XRCC1* rs1799782 is not yet well understood.

Another enzyme that plays a primary role in base excision repair is *APEX1*. *APEX1* completes the restoration of DNA damage by excising abasic residues and poly polymerase-1 binding in DNA containing strand breaks, DNA polymerase-*β*, polynucleotide kinase, and DNA ligase III. For the rs1130409 (Asp148Glu, -656T>G) polymorphism of *APEX1*, functional studies suggest that mutation to a G allele may alter endonuclease DNA-binding activity, reduce its ability to communicate with other base excision repair proteins, and decrease its capacity to repair DNA damage induced by oxidative stress [22, 23].

Several studies have reported on the adverse effects of smoking on hearing ability [24, 25]. Likewise, our results showed an interaction between cigarette use and SNPs (rs1799782 and rs1130409) with an NIHL risk of OR = 3.71. Adverse effects were also observed between alcohol consumption and NIHL in this study. However, there are still controversies regarding the effects of smoking and drinking on hearing loss. As such, further studies are required to confirm these findings [26, 27].

Our study was the first to investigate the association between the *XRCC1* rs1799782 and *APEX1* rs1130409 polymorphisms and NIHL risk. One limitation of our study was that the workers enrolled in our study were exposed to steady noise for more than 20 years but have lower levels of exposure to other occupational hazards. Moreover, the NIHL workers with both a low- and high-frequency hearing range worse than 25 dB were all transferred from noisy environments. Therefore, a selection bias may exist in our study.

## 5. Conclusion

Our findings support a potential association of the *XRCC1* rs1799782 and *APEX1* rs1130409 variants with inherited susceptibility to NIHL. However, the concrete mechanism underlying NIHL association with *XRCC1* rs1799782 and *APEX1* rs1130409 will need to be investigated in future studies.

## Figures and Tables

**Table 1:** Demographic characteristics of study subjects.

Variables	Sensitive group (*n* = 117)		Resistant group (*n* = 117)		*P*
	*n*	%	*n*	%	
Age (years)					
Mean ± SD	40.72 ± 6.60		41.87 ± 4.56		0.121^a^
Sex					
Male	112	95.7	109	93.2	0.392^b^
Female	5	4.3	8	6.8	
Tobacco use					
Now	59	50.4	61	52.1	0.249^b^
Ever	3	2.6	8	6.8	
Never	55	47.0	48	41.0	
Alcohol consumption					
Now	40	34.2	51	43.6	0.374^c^
Ever	3	2.6	3	2.6	
Never	74	63.2	2.6	53.8	
Work time with noise (years)					
Mean ± SD	19.18 ± 7.67		18.79 ± 6.94		0.288^a^
Expose level with noise (dB)					
Mean ± SD	87.01 ± 8.11		87.01 ± 6.37		1.000^a^
Hearing threshold level (dB)					
Mean ± SD	52.35 ± 6.63		8.98 ± 2.27		**<0.001** ^a^
<26	0	0	117	100.0	
≥26	117	100	0	0.0	

^a^Students' *t*-test; ^b^Two-sided *χ*^2^ test; ^c^Fisher's exact test.

**Table 2:** General information of selected SNPs and the Hardy-Weinberg test.

Gene	SNP	Alleles	Chromosome	Functional consequence	MAF		*P* for HWE^b^
					Control^a^	Database	
hOGG1	rs2072668	C/G	3 : 9756456	Intron variant	0.376	0.378	0.926
XRCC1	rs1799782	C/T	19 : 43553422	Missense	0.296	0.267	0.149
APEX1	rs1130409	G/T	14 : 20456995	Missense	0.438	0.452	0.529

^a^Data from NCBI dbSNP; ^b^*P* value of the Hardy-Weinberg test.

**Table 3:** Distribution of three polymorphisms and the association with NIHL.

Genetic models	Genotypes	Sensitive group		Resistant group		Adjusted *P*^a^	Adjusted OR (95% CI)^a^
		*n* = 117	%	*n* = 117	%		
rs2072668							
Codominant	GG	34	29.1	39	33.3		1.00 (ref.)
	CC	15	14.5	17	14.5	0.874	1.07 (0.45-2.55)
	CG	68	52.1	61	52.1	0.359	1.32 (0.73-2.38)
Dominant	GG	34	29.1	39	33.3		1.00 (ref.)
	CC+CG	83	70.9	78	66.7	0.414	1.27 (0.72-2.25)
Recessive	CG+GG	102	87.2	100	85.5		1.00 (ref.)
	CC	15	12.8	17	14.5	0.766	0.89 (0.41-1.92)
Alleles	G	136	58.1	139	59.4		1.00 (ref.)
	C	98	41.9	95	40.6	0.695	1.08 (0.74-1.57)

rs1799782							
Codominant	CC	51	43.6	59	50.4		1.00 (ref.)
	CT	52	44.4	56	47.9	0.940	1.02 (0.59-1.76)
	TT	14	12.0	2	1.7	**0.005**	8.92 (1.91-41.63)
Dominant	CC	51	43.6	59	50.4		1.00 (ref.)
	CT+TT	66	56.4	58	49.6	0.344	1.29 (0.76-2.17)
Recessive	CC+CT	103	88.0	115	98.3		1.00 (ref.)
	TT	14	12.0	2	1.7	**0.005**	8.83 (1.93-40.36)
Alleles	C	154	65.8	174	74.4		1.00 (ref.)
	T	80	34.2	60	25.6	**0.046**	1.51 (1.01-2.26)

rs1130409							
Codominant	TT	28	23.9	49	41.9		1.00 (ref.)
	GG	26	22.2	21	17.9	**0.039**	2.21 (1.04-4.70)
	GT	63	53.8	47	40.2	**0.004**	2.48 (1.34-4.61)
Dominant	TT	28	23.9	49	41.9		1.00 (ref.)
	GG+GT	89	76.1	68	58.1	**0.003**	2.39 (1.34-4.27)
Recessive	GG	26	22.2	21	17.9		1.00 (ref.)
	GT+TT	91	77.8	96	82.1	0.428	1.30 (0.68-2.51)
Alleles	T	119	50.9	145	62.0		1.00 (ref.)
	G	115	49.1	89	38.0	**0.015**	1.59 (1.10-2.31)

^a^Adjusted for age, sex, tobacco use, and alcohol consumption in the logistic regression model.

**Table 4:** Stratified analysis of SNPs in the allelic model.

SNPs	Group	Alleles	Cumulative noise exposure (dB)	
			≤95	>95
rs2072668	Sensitive group	C	17	81
		G	29	107
	Resistant group	C	46	49
		G	64	75
	Adjusted *P*^a^		0.613	0.390
	Adjusted OR (95% CI)^a^		0.83 (0.39-1.73)	1.23 (0.76-1.99)

rs1799782	Sensitive group	C	31	123
		T	15	65
	Resistant group	C	79	95
		T	31	29
	*P* ^a^		0.611	**0.034**
	Adjusted OR (95% CI)^a^		1.22 (0.57-2.63)	1.76 (1.05-2.98)

rs1130409	Sensitive group	G	20	95
		T	26	93
	Resistant group	G	38	51
		T	72	73
	*P* ^a^		0.309	0.126
	Adjusted OR (95% CI)^a^		1.46 (0.71-3.03)	1.44 (0.90-2.30)

dB: decibel; ^a^Adjusted for age, sex, tobacco use, and alcohol consumption in the logistic regression model.

**Table 5:** Analysis of the interaction by GMDR.

Best model	Training balanced accuracy	Testing balanced accuracy	Cross-validation consistency	*P*	OR (95% CI)
rs1130409	0.5897	0.5897	10/10	0.0037	2.29 (1.31-4.02)
rs1130409^∗^drink	0.6211	0.5641	7/10	0.0002	2.77 (1.61-4.77)
rs1799782^∗^rs1130409^∗^smoke	0.6629	0.5513	5/10	<0.0001	3.71 (2.16-6.38)

## Data Availability

The data used to support the findings of this study are available from the corresponding authors upon request. General characteristics of the patients are presented in Table 1.
